# Efficacy of Psychological Treatments for Patients With Schizophrenia and Relevant Negative Symptoms: A Meta-Analysis

**DOI:** 10.32872/cpe.v2i3.2899

**Published:** 2020-09-30

**Authors:** Marcel Riehle, Mara Cristine Böhl, Matthias Pillny, Tania Marie Lincoln

**Affiliations:** a Clinical Psychology and Psychotherapy, Universität Hamburg, Hamburg, Germany; Philipps-University of Marburg, Marburg, Germany

**Keywords:** schizophrenia and psychosis, negative symptoms, psychotherapy, nonpharmacological treatment, meta-analysis

## Abstract

**Background:**

Recent meta-analyses on the efficacy of psychological treatments for the negative symptoms of schizophrenia included mostly trials that had not specifically targeted negative symptoms. To gauge the efficacy of such treatments in the target patient population – namely people with schizophrenia who experience negative symptoms – we conducted a meta-analysis of controlled trials that had established an inclusion criterion for relevant negative symptom severity.

**Method:**

We conducted a systematic literature search and calculated random-effects meta-analyses for controlled post-treatment effects and for pre-post changes within treatment arms. Separate analyses were conducted for different therapeutic approaches. Our primary outcome was reduction in negative symptoms; secondary outcomes were amotivation, reduced expression, and functioning.

**Results:**

Twelve studies matched our inclusion criteria, testing Cognitive Behavioral Therapy (CBT) vs. treatment-as-usual (k = 6), Cognitive Remediation (CR) vs. treatment-as-usual (k = 2), CBT vs. CR (k = 2), and Body-oriented Psychotherapy (BPT) vs. supportive group counseling and vs. Pilates (k = 1 each). Accordingly, meta-analyses were performed for CBT vs. treatment-as-usual, CR vs. treatment-as-usual, and CBT vs. CR. CBT and CR both outperformed treatment-as-usual in reducing negative symptoms (CBT: Hedges’ g = -0.46; CR: g = -0.59). There was no difference between CBT and CR (g = 0.12). Significant pre-post changes were found for CBT, CR, and to a lesser extent for treatment-as-usual, but not for BPT.

**Conclusion:**

Although effects for some approaches are promising, more high-quality trials testing psychological treatments for negative symptoms in their target population are needed to place treatment recommendations on a sufficiently firm foundation.

## Abstract (highlights)

- This meta-analysis assesses the efficacy of psychological treatments for relevant negative symptoms.
- Cognitive Behavioral Therapy and Cognitive Remediation show promising effects reducing symptoms.
- Interventions show differential effects for the subcomponents amotivation and reduced expression.
- The evidence-base is not in line with recommendations made in treatment guidelines.

The negative symptoms of schizophrenia, i.e. blunted affect, alogia, anhedonia, asociality, and avolition (Marder & Galderisi, 2017), are among the best predictors of patients’ social functioning levels (Fervaha, Foussias, Agid, & Remington, 2014; Galderisi et al., 2014) and accordingly an important treatment target. With respect to psychological treatments, meta-analyses have reported moderate treatment effects for negative symptoms in response to cognitive behavioral therapy for psychosis (CBTp) (Wykes, Steel, Everitt, & Tarrier, 2008), cognitive remediation (CR) (Cella, Preti, Edwards, Dow, & Wykes, 2017; Roder, Mueller, & Schmidt, 2011), social skills training (SST) (Kurtz & Mueser, 2008; Turner et al., 2018), and mindfulness-based interventions (Khoury, Lecomte, Gaudiano, & Paquin, 2013). In the case of CBT, the effect was not significant in a more recent meta-analysis (Velthorst et al., 2015). Among studies comparing different active psychological interventions to one another, SST seems to be superior to other treatments (Turner, van der Gaag, Karyotaki, & Cuijpers, 2014) and is recommended for negative symptoms in two German treatment guidelines (DGPPN e.V., 2019; Lincoln, Pedersen, Hahlweg, Wiedl, & Frantz, 2019). According to the British NICE guidelines (NICE, 2014), offering arts therapy (including music and body-oriented therapy) should be considered both in acute phases and “to assist in promoting recovery, particularly in people with negative symptoms” (p. 220). NICE does not recommend any other approach for negative symptoms.

## Why yet Another Meta-Analysis?

Besides the mixed conclusions from previous meta-analyses, all of the meta-analyses mentioned share the limitation that almost all included original trials reported on negative symptoms as a secondary, not a primary outcome. For example for CBTp, only 3 out of 30 studies (Velthorst et al., 2015; Wykes et al., 2008) specifically targeted negative symptoms. In the case of CR, Cella et al. (2017), p. 43, noted that “negative symptoms have not been considered a primary target for CR”. Instead, due to the focus on positive symptoms in most included trials, participants in the trials often had passed some minimum criterion for the presence of positive symptoms. Therefore, we cannot rule out that the moderate meta-analytic effects for negative symptoms mentioned above result from primary studies that did not include any patients with relevant^1^1Because there are no unified criteria to demarcate the presence from the absence of negative symptoms, we use the concept of „relevant negative symptoms“ throughout this paper as an umbrella term for the different ways that have been put forward to describe negative symptoms that can be considered in need of treatment (see for instance Table 1 in this paper or the differing criteria used in Buchanan, 2007 and Bobes et al., 2010). negative symptoms. This makes it extremely difficult to select appropriate treatments for the patients with schizophrenia, who present with relative negative symptoms, which have been estimated to constitute one (Buchanan, 2007) or even two (Bobes, Arango, Garcia-Garcia, & Rejas, 2010) thirds of the total patient population. To emphasize this point; this is as if we wanted to judge the efficacy of an intervention for auditory hallucinations on the basis of studies that did not make sure that their participants actually had auditory hallucinations before the intervention.

More specifically, because previous meta-analyses did not limit their eligibility criteria to studies that required that their patients present with at least some relevant level of negative symptoms, there are several possible ways by which these meta-analyses may have either over- or underestimated the effect size of psychological negative symptom treatments. For instance, floor effects need to be expected if patients without relevant negative symptoms and thus little room for improvement in this domain are included in the studies. This would lead to an underestimation of the effect size. On the other hand, we need to consider the possibility that patients with more severe negative symptoms benefit less from therapy or that the interventions’ effects primarily reflect changes in the so-called “secondary” (Carpenter, Heinrichs, & Wagman, 1988) negative symptoms (e.g., social withdrawal due to paranoia). Each of these would lead to an overestimation of the effect size. In fact, at least the latter possibility is likely, given that–much more often than not–positive symptoms were the focus of the primary research that fed into the meta-analyses mentioned above. Another problem with this focus of most considered trials is that the interventions analyzed usually targeted positive psychotic symptoms and for this reason were derived from psychological models of those symptoms. Given that positive and negative symptoms are usually uncorrelated (e.g., Engel, Fritzsche, & Lincoln, 2014; Strauss et al., 2012), it is not scientifically plausible that these interventions should work well for negative symptoms.

To overcome these uncertainties, we conducted a meta-analysis of only those controlled treatment studies that focused specifically on psychological interventions for negative symptoms *and* that made sure that enrolled patients presented with relevant negative symptoms. As the primary outcome, we estimated the controlled meta-analytic effect size for negative symptoms post treatment. As secondary outcomes, we estimated the controlled meta-analytic effect size for each of the two negative symptom dimensions, motivational and expressive negative symptoms (Blanchard & Cohen, 2006), as well as for level of functioning. As a secondary analysis, we estimated the meta-analytic pre-post changes within treatment arms for each outcome.

## Method

### Eligibility Criteria

We defined six eligibility criteria in accordance with the PICOS criteria. First, we included only studies that exclusively enrolled adult *patients* with a diagnosis of schizophrenia spectrum disorder according to DSM or equivalent ICD diagnoses. Second, studies were eligible only when they had established any minimum inclusion criterion of negative symptom severity (i.e. relevant negative symptoms). Third, studies were eligible when they tested a psychological *intervention*, defined as manual-based non-invasive non-pharmacological talk- or exercise-based intervention and when this intervention specifically targeted negative symptoms. Fourth, all eligible studies had to include either a wait-list condition (e.g., treatment-as-usual, TAU) or an alternative active intervention as a *comparator*. Fifth, eligible studies needed to report *outcomes* on at least one of the following validated negative symptom assessments: Brief Negative Symptom Scale (BNSS; Kirkpatrick et al., 2011), Clinical Assessment Interview for Negative Symptoms (CAINS; Horan, Kring, Gur, Reise, & Blanchard, 2011), Negative Symptom Assessment (NSA; Alphs, Summerfelt, Lann, & Muller, 1989), Positive and Negative Syndrome Scale (PANSS; Kay, Fiszbein, & Opler, 1987), Scale for the Assessment of Negative Symptoms (SANS; Andreasen, 1989). Sixth, eligible studies had to be designed as controlled trials (CT) or randomized controlled trials (RCT). Finally, studies were only eligible if they reported on original data (i.e. no secondary analyses) and were published in a peer-reviewed journal in English or German language.

### Literature Search

We searched the databases of MEDLINE(R) and PsycINFO on August 24, 2020, using the following search term: (negative symptoms) AND (schizophrenia OR psychosis) AND (treatment OR intervention OR therapy OR psychotherapy OR training OR remediation). We also consulted reference lists of several systematic reviews and meta-analyses (Cella et al., 2017; Devoe, Peterson, & Addington, 2018; Khoury et al., 2013; Kurtz & Mueser, 2008; Lutgens, Gariepy, & Malla, 2017; Roder et al., 2011; Turner et al., 2014; Velthorst et al., 2015; Wykes et al., 2008). M.C.B. screened titles and abstracts of all studies in the search pool for non-eligibility and read full texts of all potentially eligible studies. M.C.B. made final decisions on eligible studies and resolved any uncertainties with M.R. A hierarchical decision structure was used to code the reason for exclusion of a study after reading the full-text: a) not retrievable, b) not a treatment study, c) secondary analysis, d) no CT or RCT, e) included patients outside the diagnostic spectrum, f) did not report on a validated negative symptom assessment, g) no inclusion criterion for relevant negative symptoms, h) data reported insufficiently for meta-analysis. In the case of insufficient data, we contacted the study’s corresponding author up to four times to request data.

### Data Extraction

We developed a coding protocol based on the *Cochrane Handbook* (Higgins & Deeks, 2008). The full item list can be requested from the first author.

For our primary outcome, negative symptoms, we extracted per availability the post treatment negative symptom scores (*M* and *SD*) for the experimental and control group, respectively, or the between-group effect size estimate reported post treatment. Post-treatment scores were defined as the first assessment after the termination of the intervention. If studies reported on more than one validated negative symptom assessment, we used the data from the one assessment labelled as primary outcome in the study. For all outcomes post treatment, results from intent-to-treat analyses (e.g., last observation carried forward) were prioritized over completer analyses.

For the secondary outcomes, motivational negative symptoms, expressive negative symptoms, and level of functioning, we extracted per availability post treatment scores (*M* and *SD*) or the between-group effect size estimate reported post treatment. We defined the following as potential measures of motivational negative symptoms: BNSS scales anhedonia, asociality, and avolition, CAINS scale motivation and anticipation of pleasure, SANS scales avolition-apathy and anhedonia-asociality, and PANSS items N2 and N4 (Fervaha et al., 2014; Jang et al., 2016). We defined the following as potential measures of expressive negative symptoms: BNSS scales blunted affect and alogia, CAINS scale expressive reduction, SANS scales affective flattening and alogia, and PANSS items N1, N3, N6, and G7 (Fervaha et al., 2014; Jang et al., 2016). We defined measures of level of functioning as assessments of patients’ functionality in one or more of the following areas: family, friendship and partnership, vocation, or recreation.

For our secondary analysis on pre-post changes, we also extracted pre-treatment scores (*M* and *SD*) on negative symptoms, motivational negative symptoms, expressive negative symptoms, and level of functioning or pre-post within-group effect size estimates. Pre-treatment scores were defined as the last assessment before the start of the intervention.

### Effect Size Computation at the Levels of the Individual Studies

We computed Hedges’ *g* as the mean difference between groups (experimental minus control group) divided by the pooled standard deviation (Cohen’s *d*) multiplied with a correction term (Borenstein, 2009; Hedges & Olkin, 1985). The variance of *g* was calculated according to Borenstein, Hedges, Higgins, and Rothstein (2009) (for the complete formulae see the Supplementary Materials).

For pre-post within group comparisons we calculated *g* and its variance using the formulae for pre-post changes provided in Borenstein et al. (2009) (see Supplementary Materials for complete formulae). These formulae account for the pre-post correlation of the repeated measure (cf. McGaw & Glass, 1980) that we estimated at *r* = .50 based on the pre-post correlations of studies included in this meta-analysis (see Supplementary Materials) and in line with recommendations in the literature (Lincoln, Suttner, & Nestoriuc, 2008; Smith, Glass, & Miller, 1980).

In cases in which several subscales needed to be integrated into one measure, we estimated *d* for each subscale, and computed a study-wise mean *d,* and subsequently *g*, and estimated its variance based on an integration of the variances of the subscales and their inter-correlations (Borenstein et al., 2009). If such correlations could not be obtained from the studies themselves, they were estimated from relevant literature (for details see Supplementary Materials).

We interpreted *g* ≥ 0.2 as a small effect, *g* ≥ 0.5 as a moderate effect, and *g* ≥ 0.8 as a large effect (Cohen, 1992).

### Effect Size Integration

We integrated the effect sizes using random-effects models accounting for potential heterogeneity between studies. The effect sizes of single studies were weighted by their inverse variance (Shadish & Haddock, 2009). Variance among studies was estimated according to DerSimonian and Laird (1986). We assessed heterogeneity between studies with the *Q*- and *I*^2^-statistics (Higgins, Thompson, Deeks, & Altman, 2003; Shadish & Haddock, 2009). In accordance with Higgins et al. (2003), we defined heterogeneity assessed with *I*^2^ as low (25%), moderate (50%), and high (75%). All analyses were conducted with the package *metafor* (Viechtbauer, 2010) in RStudio version 1.1.453. All significance tests were performed on an α-level of .05.

Because we were interested in comparing the efficacy of different psychological treatments for negative symptoms, we calculated separate meta-analyses for each psychological treatment approach identified in our search. Based on a recent literature review (Riehle, Pillny, & Lincoln, 2017), we expected to find studies for the following approaches: CBT, SST, CR, and body-oriented psychotherapy (BPT). We also planned to analyze studies comparing an intervention to TAU separately from studies comparing an intervention to an active control condition or an alternative treatment. We integrated effect sizes, when two or more studies were found that could be integrated.

### Risk of Bias Analyses

Risk of bias for individual studies was assessed with seven criteria that were based on the *Cochrane Risk of Bias Tool* (Higgins, Altman, & Sterne, 2008). The seven criteria were evaluated on a dichotomous true (high quality)/false (low quality) scale and were: a) use of randomization for group allocation, b) use of an intent-to-treat analysis to account for dropouts, c) assessment of treatment fidelity, d) assessors blinded to group allocation, e) non-selective reporting of outcomes, f) matching of experimental and control group, g) exclusion of patients with high levels of positive psychotic symptoms (cf. Savill, Banks, Khanom, & Priebe, 2015).

To account for potential publication bias influencing the meta-analysis, we inspected funnel plots (effect sizes plotted against their standard errors) for asymmetry (Borenstein et al., 2009; Sterne, Egger, & Moher, 2008) and conducted trim-and-fill analyses (Duval & Tweedie, 2000).

## Results

### Study Selection

**Figure 1:**
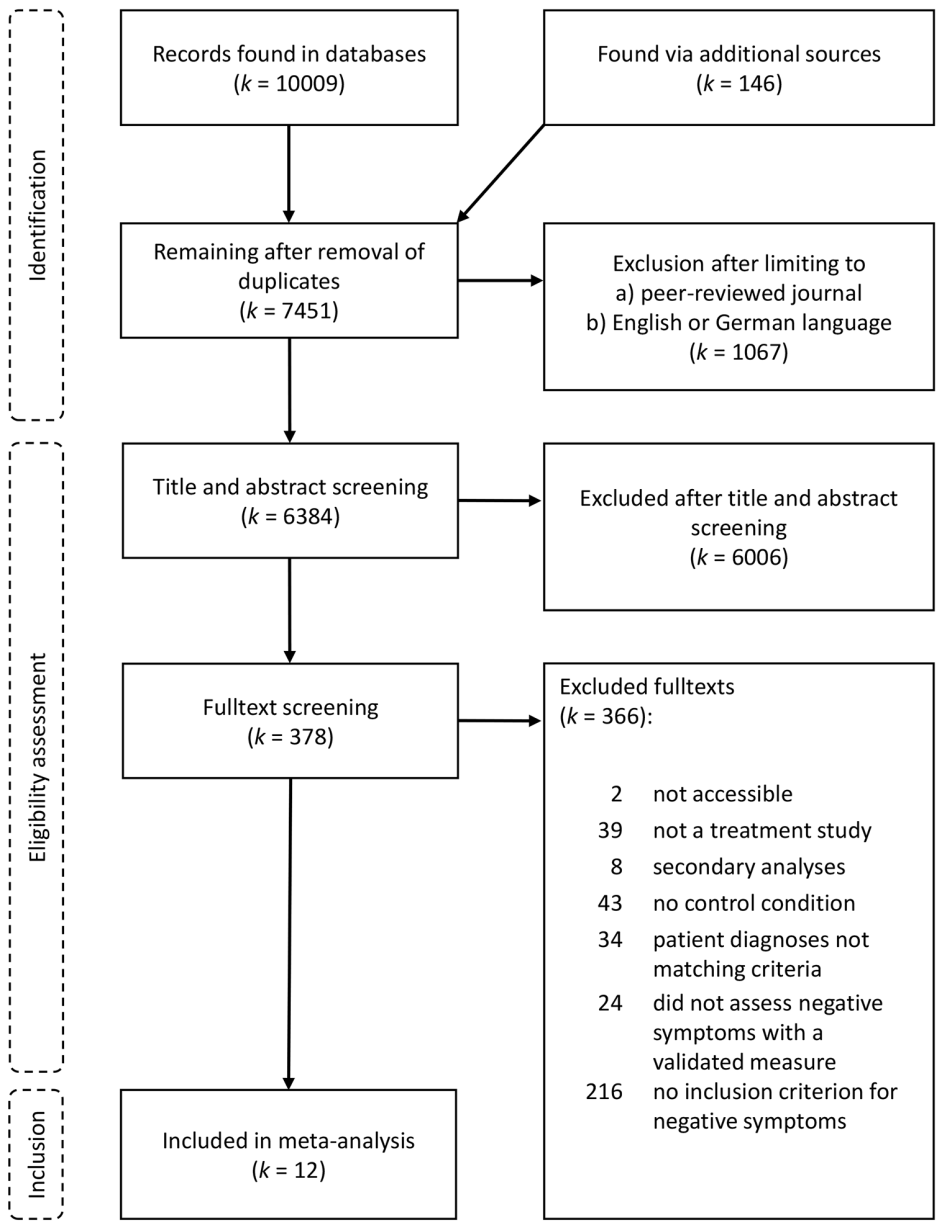
Flow Chart of the Literature Selection Process

The flow-chart in Figure 1 illustrates the study selection process. We identified *k* = 12 studies fulfilling our inclusion criteria. Of the twelve studies, *k* = 6 tested CBT vs. TAU (Bailer, Takats, & Westermeier, 2001; Choi, Jaekal, & Lee, 2016; Favrod et al., 2019; Grant, 2012; Pos et al., 2019; Velligan et al., 2015), *k* = 2 tested CBT vs. CR (Klingberg et al., 2011; Penadés et al., 2006), *k* = 2 tested CR vs. TAU (Li et al., 2019; Mueller, Khalesi, Benzing, Castiglione, & Roder, 2017), *k* = 1 tested BPT vs. group supportive counselling (Röhricht & Priebe, 2006), *k* = 1 tested BPT vs. Pilates (Priebe, Savill, Wykes, Bentall, Lauber, et al., 2016a; Priebe, Savill, Wykes, Bentall, Reininghaus, et al., 2016b).

Accordingly, we calculated meta-analyses for the comparisons of CBT vs. TAU, CR vs. TAU, and CBT vs. CR. For the meta-analysis of pre-post changes in negative symptoms within the study groups, we integrated data from all samples included in the twelve studies that received comparable forms of treatment: CBT (*k* = 8), CR (*k* = 4), BPT (*k* = 2), TAU (*k* = 8). Data was not available for all outcomes in all studies and Tables S3 and S4 in the Supplementary Materials show in detail which studies were included in which analyses.

The study characteristics are shown in Table 1. As can be seen, every study used a unique criterion to establish a minimum level of negative symptom severity.

**Table 1:** Characteristics of Studies Included in the Meta-Analysis, Sorted by Comparison

Comparison/ Reference	Country of origin	*N*^a^ EG^b^ / CG^c^	Drop-outs EG^b^/CG^c^	Male sex EG^b^/CG^c^	Treatment duration in weeks	Primary outcome measure	Mot./Exp. NES measure	Level of functioning	NES inclusion criterion
CBT vs. TAU									
Bailer et al., 2001	GER	20 / 19	13% / 21%	54% / 58%	12	SANS	SANS	DAS-M	≥ 2 on any SANS scale or DAS-M global
Choi et al., 2016	KOR	22 / 19	4% / 21%	52% / 50%	10	PANSS-N	BNSS	-	> 3 on at least 2 PANSS-N items
Favrod et al., 2019	CHE	40 / 40	8% / 0%	53% / 70%	8	SANS	SANS	-	≥ 2 on SANS apathy/anhedonia
Grant et al., 2012	USA	31 / 29	10% / 10%	68% / 66%	72	SANS	SANS	GAF	≥ 4 on at least 1 or ≥ 3 on 2 SANS scales
Pos et al., 2019	NED	49 / 50	18% / 20%	76% / 86%	10	BNSS	BNSS	GAF	PANSS N2 or N4 ≥ 3 or BNSS asociality items ≥ 2
Velligan et al., 2015	USA	17 / 22	35% / 12%	65% / 68%	36	NSA	CAINS	-	> 3 on at least 2 NSA symptom domains
CR vs. TAU									
Li et al., 2019	CHI	16 / 15	6% / 27%	53% / 72%	4	PANSS-N	SANS	-	PANSS-N at least 6 points > PANSS-P
Mueller et al., 2017	CHE	28 / 33	14% / 6%	76% / 79%	15	PANSS-N	PANSS items N1, N4, N6	GAF	> 3 on PANSS N1, N4, and/or N6
CBT vs. CR									
Klingberg et al., 2011	GER	99 / 99	9% / 20%	59% / 53%	36	PANSS-MNS	SANS	GAF	> 10 on PANSS-MNS sum score
Penadés et al., 2006	ESP	20 / 20	15% / 20%	55% / 60%	16	PANSS-N	-	LSP	PANSS-N > PANSS positive scale
BPT vs. Pilates									
Priebe et al., 2016b	GBR	131 / 123	2% / 4%	50% / 48%	10	PANSS-N	CAINS	MANSA	≥ 18 on PANSS-N
BPT vs. GSC									
Röhricht & Priebe, 2006	GBR	24 / 19	4% / 9%	74% / 74%	10	PANSS-N	PANSS items N1, N6	MANSA	≥ 20 on PANSS-N and/or ≥ 6 on PANSS N1, N2, or N6

*Note*. EG = experimental group; CG = control group; NES = negative symptoms; CBT = Cognitive Behavioral Therapy; TAU = Treatment-as-Usual; CR = Cognitive Remediation; BPT = Body-oriented Psychotherapy; GSC = Group Supportive Counselling; SANS = Scale for the Assessment of Negative Symptoms; DAS-M = Disability Assessment Schedule; PANSS-N/MNS = Positive and Negative Syndrome Scale Negative Scale/Modified Negative Factor (N1, N2, N3, N4, N6, G7, G16); BNSS = Brief Negative Symptom Scale; NSA = Negative Symptom Assessment; CAINS = Clinical Assessment Interview for Negative Symptoms; GAF = Global Assessment of Functioning; LSP = Life Skills Profile; MANSA = Manchester Short Assessment of Quality of Life.

^a^*N* corresponds to number of participants available for a meta-analysis on the primary outcome measure. ^b^CBT for CBT vs. CR. ^c^CR for CBT vs. CR.

### Controlled Post-Treatment Effects

**Figure 2:**
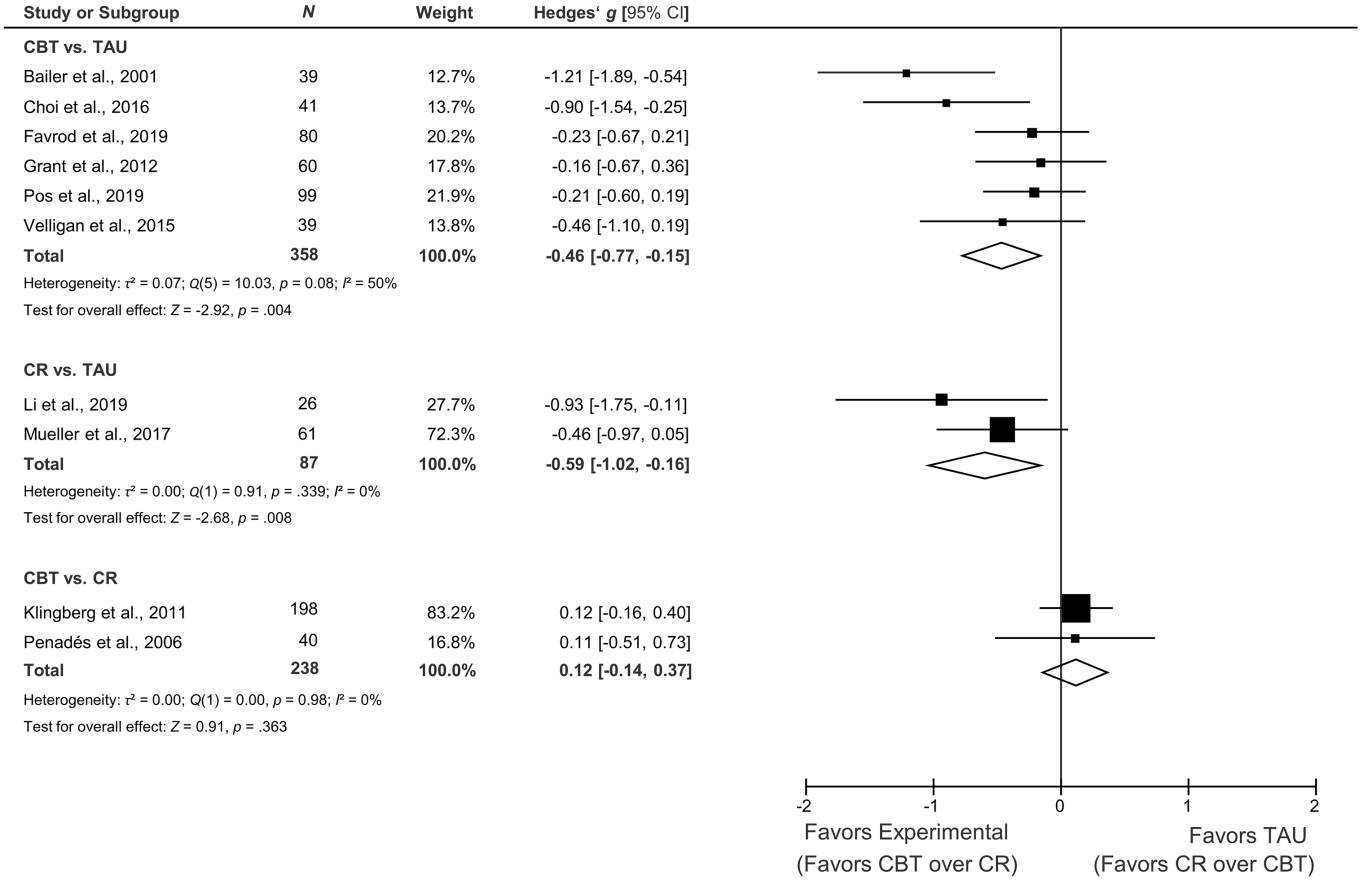
Forest Plot of the Random Effects Meta-Analyses for the Controlled Treatment Effects of CBT vs. TAU, CR vs. TAU, and CBT vs. CR in Reducing Relevant Negative Symptoms

Figure 2 contains the forest plots for the comparisons of CBT vs. TAU, CR vs. TAU and CBT vs. CR on controlled effect sizes for a global measure of negative symptoms.

#### CBT vs. TAU

As can be seen in Figure 2, there was a moderate and significant treatment effect favoring CBT over TAU for our primary outcome, negative symptoms post treatment. Heterogeneity across the four studies was moderate.

Regarding secondary outcomes, for motivational negative symptoms, there was a moderate significant post treatment effect favoring CBT over TAU *k* = 6, *N* = 347, *g* = -0.50, 95% CI [-0.77, -0.22] (heterogeneity: *Q* = 8.04, *p* = .154, *I*^2^ = 37.8%). For expressive negative symptoms, there was no difference between CBT and TAU, *k* = 5, *N* = 248, *g* = -0.05, 95% CI [-0.30, 0.20] (heterogeneity: *Q* = 4.29, *p* = .369, *I*^2^ = 6.70%). For level of functioning, there was a moderate but non-significant and highly heterogeneous effect favoring CBT over TAU, *k* = 3, *N* = 198, *g* = 0.56, 95% CI [-0.11, 1.23] (heterogeneity: Q = 9.95, *p* = .007, *I*^2^ = 79.9%).

#### CR vs. TAU

As also can be seen in Figure 2, there was a moderate and significant treatment effect favoring CR over TAU for our primary outcome, negative symptoms post treatment. No heterogeneity was noted across the two studies.

Regarding secondary outcomes, for motivational negative symptoms, there was a small but non-significant post treatment effect favoring CR over TAU *k* = 2, *N* = 87, *g* = -0.23, 95% CI [-0.64, 0.19] (heterogeneity: *Q* = 0.80, *p* = .371, *I*^2^ = 0.0%). For expressive negative symptoms, there was a moderate and significant effect favoring CR over TAU, *k* = 2, *N* = 87, *g* = -0.53, 95% CI [-0.93, -0.12] (heterogeneity: *Q* = 0.30, *p* = .584, *I*^2^ = 0.0%). For level of functioning, only one study reported sufficient data (Mueller et al., 2017), so that no effect size integration was performed.

#### CBT vs. CR

As shown in Figure 2, there was no significant difference between CBT and CR for negative symptoms post treatment and the heterogeneity measure indicated uniformity of the two studies’ effects.

Regarding the secondary outcomes, for level of functioning, there was a small but non-significant post treatment effect favoring CR over CBT, *k* = 2, *N* = 238, *g* = 0.31, 95% CI [-0.71, 1.34] with high heterogeneity, *Q* = 8.47, *p* = .004, *I*^2^ = 88.2%. For motivational and expressive negative symptoms, only one of the two studies reported sufficient data (Klingberg et al., 2011), so that no effect size integration was performed.

### Pre-Post Within Group Changes

The meta-analytic results for the pre-post within group changes are detailed in Table 2. For our primary outcome, global negative symptoms, significant moderate effects were noted for CBT and CR. The moderate effect of BPT was non-significant and highly heterogeneous. A small significant effect emerged for TAU.

**Table 2:** Results of the Random-Effects Meta-Analyses on Pre-Post Changes Within Treatment Arms for Primary and Secondary Outcomes, Sorted by Type of Intervention

Intervention	*k*	*N*	*g*	95% CI	*Q*	*I* ^2^
Global negative symptoms						
CBT	7	286	-0.50***	-0.66, -0.35	8.54	29.7%
CR	4	162	-0.60***	-0.86, -0.35	5.33	43.7%
BPT	2	154	-0.62^†^	-1.36, 0.11	7.93**	87.4%
TAU	7	194	-0.20*	-0.38, -0.03	8.74	31.3%
Motivational negative symptoms						
CBT	7	289	-0.58***	-0.90, -0.26	36.92***	83.8%
CR	3	142	-0.59*	-1.11, -0.07	11.69**	82.9%
BPT	-	-	-	-	-	-
TAU	8	220	-0.26**	-0.45, -0.06	14.09*	50.3%
Expressive negative symptoms						
CBT	5	209	-0.24**	-0.41, -0.08	5.43	26.4%
CR	3	142	-0.48***	-0.64, -0.32	0.86	0.0%
BPT	2	154	-0.57	-1.41, 0.23	10.38**	90.4%
TAU	6	144	-0.10	-0.26, 0.06	4.59	0.0%
Level of functioning						
CBT	5	238	0.61***	0.30, 0.92	17.37**	77.0%
CR	3	147	0.40***	0.10, 0.70	4.63^†^	56.8%
BPT	2	152	0.10	-0.07, 0.25	0.23	0.0%
TAU	3	112	0.41*	0.08, 0.74	5.61^†^	64.3%

*Note.* CBT = Cognitive Behavioral Therapy; CR = Cognitive Remediation; BPT = Body-Oriented Psychotherapy; TAU = Treatment-as-Usual.

^†^*p* < .10. *p < .05. **p < .01. ***p < .001.

For our secondary outcome motivational negative symptoms, CBT and CR showed moderate significant effects accompanied by high heterogeneity. TAU showed a small significant effect. For BPT there was insufficient data.

For expressive negative symptoms, CR showed a significant moderate effect. A small significant effect emerged for CBT. There was also a moderate effect of BPT on expressive negative symptoms, which was, however, non-significant due to high heterogeneity. We did not find an effect of TAU.

For level of functioning, small to moderate significant effects emerged for CBT, CR, and TAU, all with moderate heterogeneity, whereas there was no effect of BPT.

### Risk of Bias Analyses

#### Publication Bias

Inspection of the funnel plots (cf. Supplementary Materials) for the three comparisons of CBT vs. TAU, CR vs. TAU, and CBT vs. CR and trim-and-fill analyses suggested the following: No studies were estimated to be missing for CBT vs. TAU and CR vs. TAU. For CBT vs. CR, one study was estimated to be missing; the corrected effect, *k* = 3, *g* = 0.12, 95% CI [-0.12, 0.36], did not change the interpretation that there was no difference between the two interventions.

#### Risk of Bias in Individual Studies and Sensitivity Analyses

The results of the quality assessment of individual studies are shown in Table 3.

**Table 3:** Results of the Quality Assessment of Included Studies, Sorted by Comparison

Comparison/ Reference	Randomization	Intent-to-treat analysis	Assessment of treatment fidelity	Blinded assessors	Non-selective outcome report	Matching groups	High levels of positive symptoms excluded
CBT vs. TAU							
Bailer et al., 2001	**-**	**-**	**-**	**-**	**+**	**+**	**-/+**
Choi et al., 2016	**-**	**-**	**+**	**-**	**+**	**+**	**+**
Favrod et al., 2019	**+**	**+**	**-/+**	**+**	**+**	**+**	**-**
Grant et al., 2012	**+**	**+**	**-**	**+**	**+**	**+**	**+**
Pos et al., 2019	**+**	**+**	**+**	**+**	**+**	**+**	**-/+**
Velligan et al., 2015	**+**	**-**	**+**	**+**	**+**	**+**	**+**
CR vs. TAU							
Li et al., 2019	**-**	**-**	**-**	**+**	**+**	**+/-**	**+**
Mueller et al., 2017	**+**	**+**	**-**	**+**	**+**	**+**	**-**
CBT vs. CR							
Klingberg et al., 2011	**+**	**+**	**+**	**+**	**+**	**+**	**+**
Penadés et al., 2006	**+**	**+**	**-**	**+**	**+**	**+**	**-/+**
BPT vs. Pilates							
Priebe et al., 2016b	**+**	**+**	**+**	**+**	**+**	**+**	**-**
BPT vs. GSC							
Röhricht & Priebe, 2006	**+**	**+**	**+**	**+**	**+**	**+**	**-**

*Note*. CBT = Cognitive Behavioral Therapy; TAU = Treatment-as-Usual; CR = Cognitive Remediation; BPT = Body-oriented Psychotherapy; GSC = Group supportive counselling; + = criterion fulfilled; - = criterion not fulfilled; -/+ = unclear; criterion probably fulfilled.

As can be seen there, the overall study quality was high. Non-selective reporting of results was implemented in all studies included in the meta-analysis and all investigated at least largely matching experimental and control groups. About half of the studies included a criterion to confine positive symptom severity in addition to their negative symptom inclusion criterion.

Three studies did not randomize their participants to the treatment arms (i.e., Bailer et al., 2001; Choi et al., 2016; Li et al., 2019). As can be seen in Figure 2, these three studies contributed the three largest controlled effect sizes. This could be due to patient preferences playing a role in group allocation (e.g., in Li et al., 2019). Also, these three studies on average fulfilled two quality criteria less than the RCTs. For this reason, we performed sensitivity analyses for all effects including only RCTs. Because for CR vs. TAU there was only a single RCT and because both CBT vs. CR and both BPT studies were RCTs, sensitivity analyses of controlled post treatment effects were performed exclusively for CBT vs. TAU. For the primary outcome, global negative symptoms, there remained a small marginally significant effect favoring CBT over TAU *k* = 4, *N* = 278, *g* = -0.24, 95% CI [-0.47, 0.004] (heterogeneity: *Q* = 0.56, *p* = .905, *I*^2^ = 0.0%). Regarding secondary outcomes, for motivational negative symptoms, there remained a small significant effect favoring CBT over TAU *k* = 4, *N* = 278, *g* = -0.35, 95% CI [-0.58, -0.11] (heterogeneity: *Q* = 1.93, *p* = .586, *I*^2^ = 0.0%). For expressive negative symptoms, there was no difference between CBT and TAU, *k* = 3, *N* = 179, *g* = 0.10, 95% CI [-0.18, 0.38] (heterogeneity: *Q* = 0.65, *p* = .723, *I*^2^ = 0.00%). Finally, for level of functioning, there remained a small but non-significant effect favoring CBT over TAU, *k* = 2, *N* = 159, *g* = 0.26, 95% CI [-0.27, 0.78] (heterogeneity: Q = 2.61, *p* = .106, *I*^2^ = 61.7%). Results of the sensitivity analyses for the pre-post effects for CBT, CR, and TAU can be found in Table S5 in the Supplementary Materials.

## Discussion

Different national treatment guidelines have recommended different psychological therapies to treat the negative symptoms of schizophrenia (e.g., DGPPN e.V., 2019; Lincoln et al., 2019; NICE, 2014). The purpose of such recommendations is to inform clinicians about which treatments to offer to their patients who experience these symptoms (i.e. the target population of the treatment). For this reason, it is important to base the recommendations on research that can answer the question whether a given treatment reduces negative symptoms in the target patient population. Here, we conducted the first systematic literature search and meta-analysis of controlled trials of psychological treatments that had employed an inclusion criterion for negative symptom severity.

Our search identified twelve controlled studies matching our inclusion criteria. These twelve studies targeted cognitive behavioral therapy (CBT), cognitive remediation (CR), and body-oriented psychotherapy (BPT). By integrating findings of studies that investigated comparable forms of treatments (e.g., all trials testing CBT vs. treatment-as-usual, TAU), we were able to calculate meta-analyses on the controlled treatment effects for the comparisons of CBT vs. TAU, CR vs. TAU, and CBT vs. CR, respectively.

We found that CBT reduced negative symptoms more than TAU with a small to moderate effect size (*g* = -0.46). This effect was larger than in other recent meta-analyses on the efficacy of CBT on negative symptoms (i.e. -0.09 to -0.16, Velthorst et al., 2015; -0.34, Lutgens et al., 2017). However, our sensitivity analysis including only RCTs suggested that the effect size could be only half as big (*g* = -0.24) in more rigorous trials. This confirms what has already been observed for CBT in psychosis more generally, namely that effect sizes tend to be smaller in more rigorous trials (Jauhar et al., 2014; Wykes et al., 2008). Having this caveat in mind, further high-quality RCTs on the efficacy of CBT for negative symptoms in the target patient population are needed to confirm (or disconfirm) the effect found in this meta-analysis.

We also found CR to reduce negative symptoms more than TAU with a moderate effect size (*g* = -0.59). Again, this effect size is considerably larger than the ones found in previous meta-analyses (i.e., Cella et al., 2017; ES = -0.30 to -0.40). However, this effect is based on only two studies, of which one (Li et al., 2019) did not randomize patients to the treatment arms and even based their treatment allocation on patients’ preferences. The only RCT that compared CR to TAU in patients with relevant negative symptoms found a moderate effect favoring CR (Mueller et al., 2017).

The similar effect sizes for CBT vs. TAU (-0.46) and CR vs. TAU (-0.59) along with the finding of no significant difference between CBT and CR suggest that CBT and CR may be similarly efficacious. As no alternative psychological treatments have been investigated for this target population compared to CBT and CR, at present we can only conclude that adding a specific psychological treatment for negative symptoms (in this case CBT or CR) to standard care reduces relevant negative symptoms more than standard care alone.

Nevertheless, the findings from our secondary outcome analyses suggest at least some degree of specificity of treatment effects for CBT and CR. For example, CBT but not CR was efficacious in reducing amotivation. In contrast, CR but not CBT had an effect on reduced expression. Moreover, as will be discussed below, BPT could be specifically efficacious to improve reduced expression but might not have an effect on amotivation. Even though these findings are certainly tentative, they highlight that there may be treatments that are specifically efficacious for the different subcomponents of negative symptoms. Therefore, future research should account for the distinction of the negative symptom subcomponents more explicitly and make these subcomponents the primary outcomes. Two of the more recent studies in our meta-analysis already adopted this approach (Favrod et al., 2019; Pos et al., 2019).

An important question then is, whether our findings accord with published treatment guidelines. For example, based on previous RCTs and meta-analyses in schizophrenia samples (e.g., Granholm, Holden, Link, & McQuaid, 2014; Kurtz & Mueser, 2008; Turner et al., 2018, 2014), the German treatment guidelines (DGPPN e.V., 2019; Lincoln et al., 2019) recommend Social Skills Training (SST) for negative symptoms. As we did not identify any study that tested SST in the target group, we argue that there is little evidence to support this recommendation. Therefore, methodologically rigorous tests of SST in patients with relevant negative symptoms are needed. In this regard, it is promising that we found one registered RCT testing Cognitive Behavioral Social Skills Training in people with relevant negative symptoms (Twamley, Granholm, & ClinicalTrials.gov, 2014).

The case of BPT, as for example recommended in the British NICE guidelines (NICE, 2014) is more complex. In our synthesis, we did not find clear evidence that BPT reduces negative symptoms. One important reason is that the large and methodologically rigorous BPT trial that we included and which was published after the last update of the NICE guidelines (Priebe et al., 2016b) mostly did not show significant results. Nevertheless, in line with other trials on BPT (Martin, Koch, Hirjak, & Fuchs, 2016; Röhricht & Priebe, 2006), the Priebe et al. (2016b) study found a significant effect for the reduction of expressive negative symptoms that did not show up in our meta-analysis for methodological reasons (i.e. the effect in Priebe et al. (2016b) only showed up as a time by group interaction). In the light of very limited treatment options for the expressive subcomponents of negative symptoms, BPT should be further explored as one potentially specific approach for this aspect of negative symptoms.

Another result of our meta-analysis is that we found a small albeit significant effect for TAU on global negative symptoms from pre to post treatment (*k* = 7, *g* = -0.20). This somewhat confirms a recent meta-analysis by Savill et al. (2015), who showed that negative symptoms decline over time in TAU conditions with a less than small, yet significant, effect (*k* = 15, ES = -0.15). Together, these findings suggest that current routine care has a negligible impact on relevant negative symptoms.

Several strengths and limitations need mentioning. Due to space restrictions, we have provided a detailed discussion of these issues in the Supplementary Materials. The limitations discussed include the heterogeneity across primary studies regarding negative symptom assessments and the negative symptom inclusion criteria. We also address the potential lack of fit between interventions and current etiological models of negative symptoms. Finally, we address strengths and limitations that arise from our strict inclusion criterion that primary studies needed to have employed an entry criterion for negative symptom severity. This includes a discussion of power issues due to the small number of primary studies. We also address how our study relates to the issue of “pseudo-specificity” in research on negative symptom treatments (cf., Fusar-Poli et al., 2015).

Having these caveats in mind, this meta-analysis indicates that routine care has a negligible effect on negative symptoms, whereas there is some evidence for the efficacy of CBT and CR. However, the effects were instable (especially for CBT) and the effect sizes leave room for improvement. Additionally, some approaches may be more promising to reduce motivational negative symptoms (CBT) and some more promising to reduce expressive negative symptoms (CR, BPT). Therefore, research efforts should be held up for the targeted and symptom-specific psychological approaches to reduce negative symptoms in order to place treatment recommendations on a firmer foundation.

## Supplementary Materials

The supplementary material contains formulae used for the calculation of effect sizes, additional results, and an in-depth discussion of strengths and limitations (for access see Index of Supplementary Materials below).

10.23668/psycharchives.3482 Supplement 1 Supplementary materials to: Efficacy of psychological treatments for patients with schizophrenia and relevant negative symptoms: A meta-analysis

RiehleM.
BöhlM. C.
PillnyM.
LincolnT. M.
 (2020). Supplementary materials to "Efficacy of psychological treatments for patients with schizophrenia and relevant negative symptoms: A meta-analysis"
 [Formulae, additional results, and discussion]. PsychOpen. 10.23668/psycharchives.3482PMC964547636398145
