# Supplementary material for: Efficacy of Psychological Treatments for Patients With Schizophrenia and Relevant Negative Symptoms: A Meta-Analysis
Source: Clin Psychol Eur. 2020 Sep 30;2(3):e2899. doi: 10.32872/cpe.v2i3.2899 (PMC9645476; doi:10.32872/cpe.v2i3.2899)
Supplement: Supplement 1 [file cpe-02-2899-s1.pdf]

Efficacy of Psychological Treatments for Patients with Schizophrenia and Relevant Negative Symptoms: A Meta-Analysis

Marcel Riehle[a*], Mara Cristine Böhl[a], Matthias Pillny[a], & Tania Marie Lincoln[a]

[a] Clinical Psychology and Psychotherapy, Universität Hamburg, Hamburg, Germany

*To whom correspondence should be addressed: marcel.riehle@uni-hamburg.de

– Supplementary Material –

Contents:

Calculation of *Hedge's g* for Controlled Post Treatment Effects 3

Calculation of *Hedges' g* for Pre-Post Within Group Changes 3

Calculation of *Hedges' g* Based on an Integration of Multiple Outcomes (Subscales) 6

Table S3. *Overview of Data Availability for Each Study and Each Secondary Outcome for Controlled Post Treatment Effects.* 8

Table S4. Overview of Data Availability for Each Study and Each Outcome for Pre-Post Within Group Changes. 9

Figure S1. Funnel plot for CBT vs. TAU. No studies to be filled in suggested by trim and fill analysis. 11

Figure S2. Funnel plot for CR vs. TAU. No studies to be filled in suggested by trim and fill analysis. 11

Figure S3. Funnel plot for CBT vs. CR including one study filled in by trim and fill analysis. For the legend, see Figure S1. 12

Table S5. Results of the Sensitivity Analyses of the Random-Effects Meta-Analyses on Pre-Post Changes Within Treatment Arms for Primary and Secondary Outcomes, Sorted by Type of Intervention. These Sensitivity Analyses Included Only RCTs. ... 13

Supplementary Discussion of Limitations and Strengths... 14

Calculation of *Hedge's g* for Controlled Post Treatment Effects

Calculation of *g* was a two-step process; (1) calculating *Cohen's d* and its variance (Var_d) and (2) adjusting both with a correction term to receive *g* and its variance (Var_g). We used the following formulae (cf. Borenstein, Hedges, Higgins, & Rothstein, 2009):

(1)

$$d = \frac{\bar{X}_{treatment,post} - \bar{X}_{control,post}}{SD_{pooled}}$$

with

$$SD_{pooled} = \sqrt{\frac{(n_{treatment}-1)\cdot SD^2_{treatment}+(n_{control}-1)\cdot SD^2_{control}}{n_{treatment}+n_{control}-2}},$$

and

$$Var_d = \frac{n_{treatment}+n_{control}}{n_{treatment}\cdot n_{control}} + \frac{d^2}{2(n_{treatment}+n_{control})}.$$

(2)

$$g = d \cdot \left(1 - \frac{3}{4df-1}\right),$$

and

$$Var_g = Var_d \cdot \left(1 - \frac{3}{4df-1}\right),$$

with

$$df = n_{treatment} + n_{control} - 2.$$

Calculation of *Hedges' g* for Pre-Post Within Group Changes

This calculation was, again, a two-step process involving (1) the calculation of *d* and Var_d, which then was (2) corrected to receive *g* and Var_g. We used the formulae provided by Borenstein et al. (2009) for pre-post effect size analyses:

(1)

$$d = \frac{\bar{X}_{post} - \bar{X}_{pre}}{SD_{pooled|pre,post}}$$

with

$$SD_{pooled} = \frac{SD_{diff|pre,post}}{\sqrt{2(1-r_{pre,post})}},$$

and with

$$SD_{diff|pre,post} = \sqrt{SD_{pre}^2 + SD_{post}^2 - 2 \cdot r_{pre,post} \cdot s_{pre} \cdot s_{post}},$$

and

$$Var_d = (\frac{1}{n_{pre}} + \frac{d^2}{2n_{pre}}) \cdot 2(1 - r_{pre,post}).$$

(2)

$$g = d \cdot \left(1 - \frac{3}{4df-1}\right),$$

and

$$Var_g = Var_d \cdot \left(1 - \frac{3}{4df-1}\right),$$

with

$$df = n_{pre} - 1.$$

The formulae in (1) require the pre-post correlation ($r_{pre,post}$) that is seldomly reported in clinical studies. Only one of the nine studies eligible for analysis reported data that was suited to infer pre-post correlations. This study, Klingberg et al. (2011), reported *Cohen's* d_z alongside means and standard deviations for pre and post assessments. We were thus able to calculate

$$r_{pre,post} = \frac{SD_{pre}^2 + SD_{post}^2 - SD_{diff|pre,post}^2}{2 \cdot SD_{pre} \cdot SD_{post}},$$

with

$$SD_{diff|pre,post} = \frac{\bar{X}_{post} - \bar{X}_{pre}}{d_z}$$

(cf. [28,30]).

Table S1 shows the correlations that emerged for the 12-month pre-to-post interval in the Klingberg et al. study. As can be seen in this table, the correlations hovered around r≈.50. In the calculations of pre-post effect sizes of the Klingberg et al. study we used the original correlations shown Table S1. For all other studies, we used $r_{pre,post}$=.50.

Table S1. *Pre-Post Correlations Calculated from the Data in the Klingberg et al. (2011) study.*

Measure	$r_{pre,post}$ CBT	CR	Mean CBT/CR
PANSS negative	0.36	0.73	0.56
SANS affective blunting	0.46	0.36	0.41
SANS alogia	0.55	0.50	0.53
SANS apathy	0.65	0.36	0.51
SANS anhedonia	0.38	0.85	0.62
SANS total	0.46	0.70	0.58
GAF	0.52	0.51	0.52

Note: CBT = Cognitive Behavioral Therapy; CR = Cognitive Remediation; PANSS = Positive and Negative Syndrome Scale; SANS = Scale for the Assessment of Negative Symptoms; GAF = Global Assessment of Functioning.

Calculation of *Hedges' g* Based on an Integration of Multiple Outcomes (Subscales)

In several cases, we had to integrate several subscales into a composite measure, because the composite measure was not available in the studies. This became necessary, for example, when estimating a composite score of motivational negative symptoms based on an integration of the SANS subscales anhedonia-asociality and avolition-apathy. We will here only present the formulae used to calculate *d*, because the respective formulae to calculate *g* from *d* are given above. Again, this approach was taken from Borenstein et al. (2009) and presumes that the formulae above were already used to calculate *d* and Var_d for each subscale to be integrated.

The integrated *d* is given by

$$d_{integrated} = \frac{\sum_{i=1}^{m} d_i}{m},$$

with

$$m = number\ of\ subscales\ to\ be\ integrated,$$

and

$$d_i = d\ of\ given\ subscale.$$

The variance of the integrated *d* is then given by

$$Var_{d,integrated} = \frac{1}{4}\sum_{i=1}^{m} Var_{d_i} + 2r_{subscales}\prod_{i=1}^{m}\sqrt{Var_{d_i}}.$$

We estimated the r between subscales based on relevant literature. Table S2 gives an overview of these correlations.

Table S2. *Overview of Correlation Coefficients Taken from Relevant Literature for the Integration of Subscales. Only Those Correlations That Were Applied in the Meta-Analysis are Shown.*

**Integrated Scale**	**Assessment**	**Subscale**	***r***	**Reference**
Negative Symptoms (composite)	SANS	Affective flattening<br>Alogia<br>Anhedonia-Asociality<br>Avolition-Apathy	.59	Peralta & Cuesta (1995)
Motivational Negative Symptoms	SANS	Anhedonia-Asociality<br>Avolition-Apathy	.69	Peralta & Cuesta (1995)
Expressive Negative Symptoms	SANS	Affective Flattening<br>Alogia	.76	Peralta & Cuesta (1995)
	PANSS	N1 Blunted Affect<br>N6 Lack of Spontaneity	.58	Riehle et al. (2015)
	PANSS	N1 Blunted Affect<br>G7 Motor Retardation	.66	Riehle et al. (2015)

Note: SANS=Scale for the Assessment of Negative Symptoms; PANSS = Positive and Negative Syndrome Scale.

Table S3. *Overview of Data Availability for Each Study and Each Secondary Outcome for Controlled Post Treatment Effects.*

	Outcome		
**Comparison/ Reference**	**Motivational Negative Symptoms**	**Expressive Negative Symptoms**	**Level of Functioning**
**CBT vs. TAU**			
Bailer 2001	**+**	**+**	**+**
Choi 2016	**+**	**+**	**-**
Favrod 2019	**+**	**+**	**-**
Grant 2012	**+**	**+**	**+**
Pos 2019	**+**	**-**	**+**
Velligan 2015	**+**	**+**	**-**
***k* for meta-analysis**	**6**	**5**	**3**
**CR vs. TAU**			
Li 2019	**+**	**+**	**- ***
Mueller 2017	**+**	**+**	**+ ***
***k* for meta-analysis**	**2**	**2**	**1**
**CBT vs. CR**			
Klingberg 2011	**+ ***	**+ ***	**+**
Penadés 2006	**- ***	**- ***	**+**
***k* for meta-analysis**	**1**	**1**	**2**

Note: Data availability is equivalent to inclusion in the respective meta-analysis, whenever the analysis could be performed. CBT=Cognitive Behavioral Therapy; TAU=Treatment-as-Usual; CR=Cognitive Remediation. + = data available, - = no data available; * = no analysis performed.

Table S4. Overview of Data Availability for Each Study and Each Outcome for Pre-Post Within Group Changes.

	CBT				CR				BPT				TAU			
**Comparison/ Reference**	**NES**	**MOT**	**EXP**	**FUNC**	**NES**	**MOT**	**EXP**	**FUNC**	**NES**	**MOT**	**EXP**	**FUNC**	**NES**	**MOT**	**EXP**	**FUNC**
**CBT vs. TAU**																
Bailer 2001	+ a)	+ a)	+ a)	+ a)	-	-	-	-	-	-	-	-	+	+	+	-
Choi 2016	+	+	+	-	-	-	-	-	-	-	-	-	+	+	+	-
Favrod 2019	+	+	+	-	-	-	-	-	-	-	-	-	+	+	+	-
Grant 2012	-	+	-	+	-	-	-	-	-	-	-	-	-	+	-	+
Pos 2019	+	+	-	+	-	-	-	-	-	-	-	-	+	+	-	+
Velligan 2015	+	+	+	-	-	-	-	-	-	-	-	-	+	+	+	-
**CR vs.TAU**																
Li 2019	-	-	-	-	+	+	+	-	-	-	-	-	+	+	+	-
Mueller 2017	-	-	-	-	+	+	+	+	-	-	-	-	+	+	+	+
**CBT vs. CR**																
Klingberg 2011	+	+	+	+	+	+	+	+	-	-	-	-	-	-	-	-
Penadés 2006	+	-	-	+	+	-	-	+	-	-	-	-	-	-	-	-
**BPT vs. Pilates**																
Priebe 2016	-	-	-	-	-	-	-	-	+	+ *	+	+	-	-	-	-
**BPT vs. GSC**																
Röhricht 2006	-	-	-	-	-	-	-	-	+	- *	+	+	-	-	-	-
***k* for meta-analysis**	**7**	**7**	**7**	**6**	**4**	**3**	**3**	**3**	**2**	**1 ***	**2**	**2**	**7**	**8**	**6**	**3**

Note: Data availability is equivalent to inclusion in the respective meta-analysis, whenever the analysis could be performed. CBT=Cognitive Behavioral Therapy; TAU=Treatment-as-Usual; CR=Cognitive Remediation; BPT=Body Oriented Psychotherapy; GSC=Group Supportice Care; NES=Negative Symptoms (composite); MOT=Motivational Negative Symptoms; EXP=Expressive Negative Symptoms; FUNC=Level of Functioning. + = data available, - = no data available; * = no analysis performed.

[a)] Combined treatment group and cross-over control group (*N* = 39) that received treatment later on as reported in (Bailer, Takats, & Schmitt, 2002).

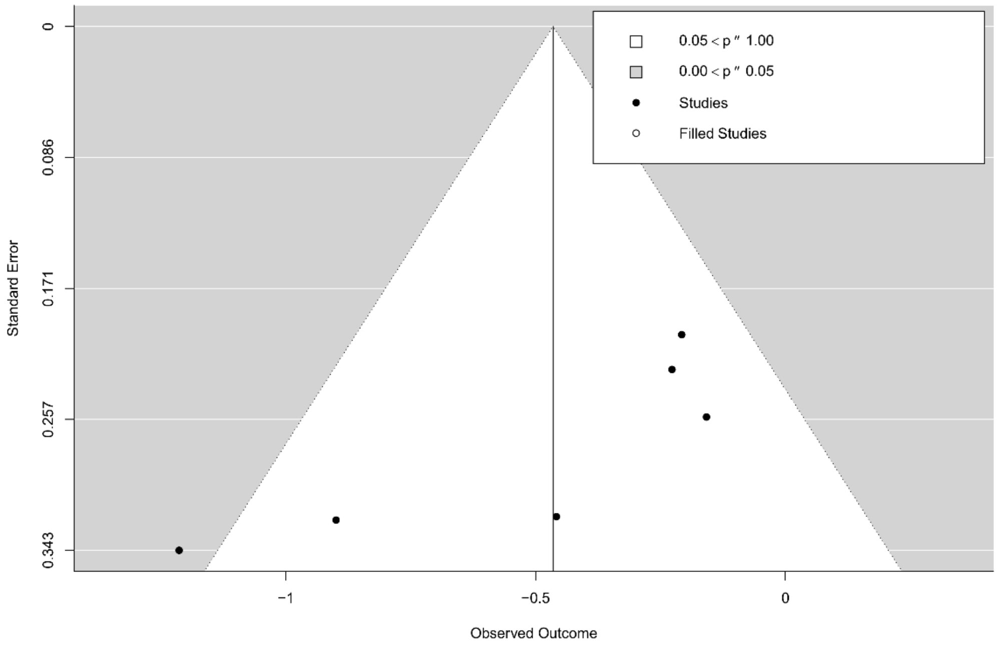

Figure S1. Funnel plot for CBT vs. TAU. No studies to be filled in suggested by trim and fill analysis.

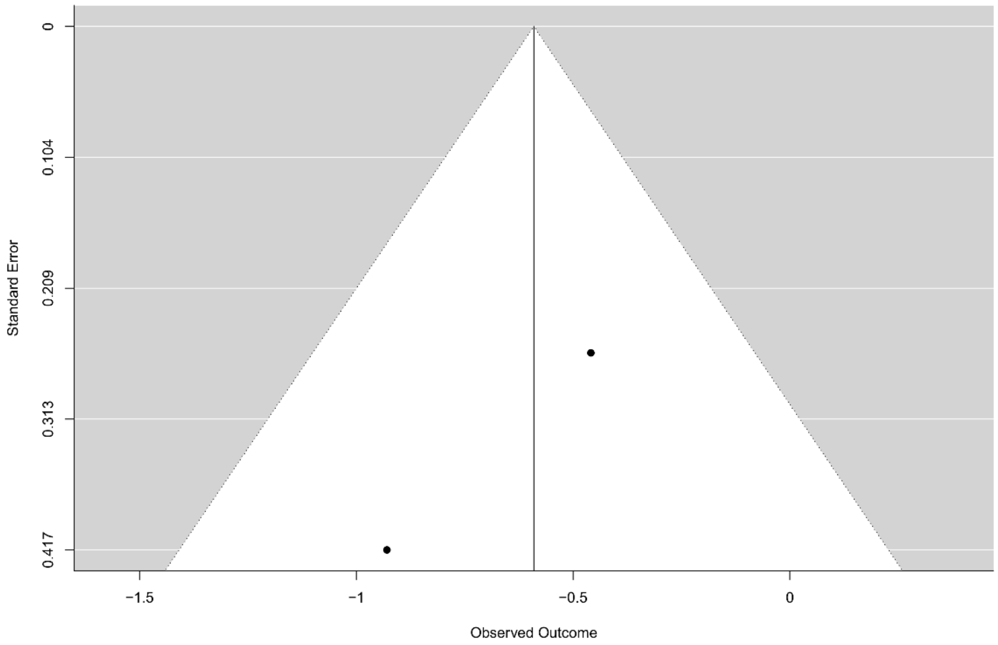

Figure S2. Funnel plot for CR vs. TAU. No studies to be filled in suggested by trim and fill analysis.

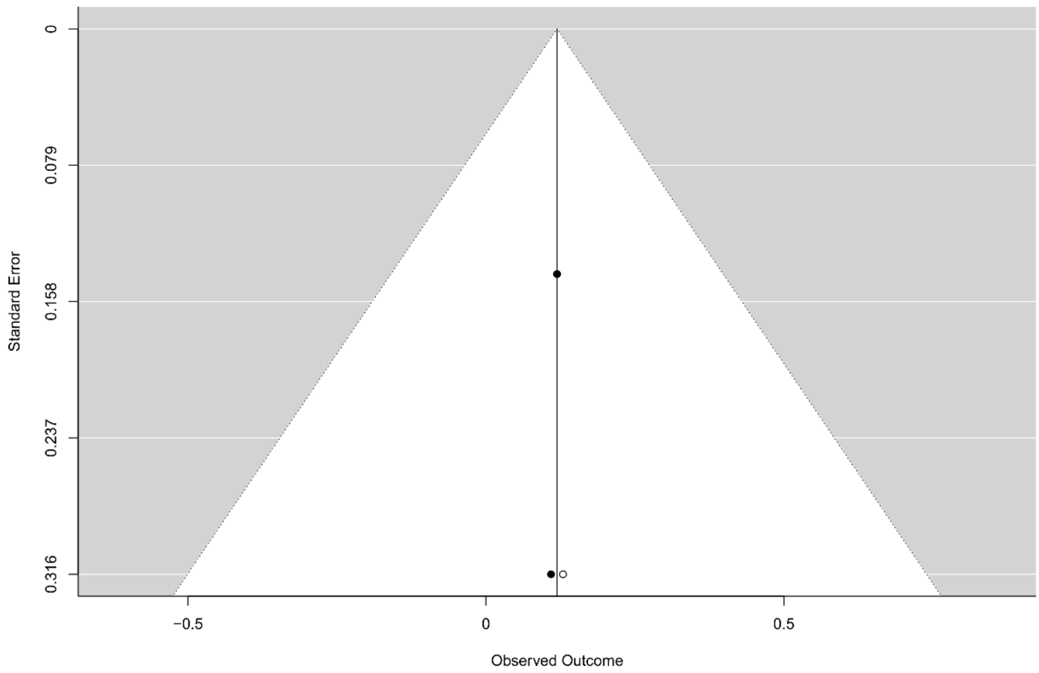

Figure S3. Funnel plot for CBT vs. CR including one study filled in by trim and fill analysis. For the legend, see Figure S1.

Table S5. Results of the Sensitivity Analyses of the Random-Effects Meta-Analyses on Pre-Post Changes Within Treatment Arms for Primary and Secondary Outcomes, Sorted by Type of Intervention. These Sensitivity Analyses Included Only RCTs.

**Intervention**	***k***	***N***	***g***	***95% CI***	***Q***	***I^2***
**Global negative symptoms**						
CBT	5	225	-0.43 ***	-0.56, -0.29	3.46	0.0%
CR	3	147	-0.55 **	-0.83, -0.27	3.85	48.1%
BPT	*2*	*154*	*-0.63* †	*-1.35, 0.08*	*7.78 ** *	*87.1%*
TAU	4	145	-0.25 **	-0.44, -0.06	3.88	22.8%
**Motivational negative symptoms**						
CBT	5	236	-0.59 **	-1.02, -0.16	33.95	88.2%
CR	2	127	-0.50	-1.16, 0.16	8.01 *	87.5%
BPT	-	-	-	-	-	-
TAU	5	174	-0.31 ***	-0.48, -0.15	4.78	16.3%
**Expressive negative symptoms**						
CBT	3	156	-0.17	-0.43, 0.10	4.74 †	57.8%
CR	2	127	-0.46 ***	-0.62, -0.29	0.27	0.0%
BPT	*2*	*154*	*-0.57*	*-1.41, 0.23*	*10.38 ** *	*90.4%*
TAU	3	95	-0.12	-0.31, 0.07	1.00	0.0%
**Level of functioning**						
CBT	4	199	0.59 **	0.21, 0.98	15.95 **	81.2%
CR	*3*	*147*	*0.40 *** *	*0.10, 0.70*	*4.63* †	*56.8%*
BPT	*2*	*152*	*0.10*	*-0.07, 0.25*	*0.23*	*0.0%*
TAU	*3*	*112*	*0.41 * *	*0.08, 0.74*	*5.61* †	*64.3%*

Note: CBT = Cognitive Behavioral Therapy, CR = Cognitive Remediation, BPT = Body-Oriented Psychotherapy, TAU = Treatment-as-Usual. Italics indicate rows where no changes had to be made to the main analyses because all included studies already were RCTs.

*** $p < .001$; ** $p < .01$; * $p < .05$; † $p < .10$.

Supplementary Discussion of Limitations and Strengths

We found that there was considerable heterogeneity across studies with respect to the definition of what we labelled "relevant" negative symptoms. In fact, all twelve studies included in our meta-analyses used a different criterion to establish relevant negative symptoms. This heterogeneity limits the generalizability of our findings and renews calls for unified criteria to establish relevant negative symptoms in clinical trials (Buchanan, 2007; Kirkpatrick, Fenton, Carpenter, & Marder, 2006; Marder & Galderisi, 2017).

A related issue is that across studies negative symptoms were measured with various scales that each have a different composition of symptoms and that have been shown to be differentially change sensitive (Savill, Banks, Khanom, & Priebe, 2015). More specifically, seven out of twelve studies relied on the PANSS, three studies on the SANS, one study on the NSA, and one study on the BNSS as their primary outcome measures and their measures to establish the negative symptom inclusion criterion. Updated assessments, the CAINS (Horan, Kring, Gur, Reise, & Blanchard, 2011) and the BNSS (Kirkpatrick et al., 2010) have been developed in order to circumvent the shortcomings of older negative symptom assessments such as the PANSS, SANS, or NSA, such as overreliance on reduced expression (e.g., PANSS) or inclusion of neuropsychological or disorganization symptoms (e.g., PANNS and SANS). The newer assessments also differentiate more clearly between motivational and expressive negative symptoms and even more fine grained symptom factors (Strauss et al., 2018). Four (Choi, Jaekal, & Lee, 2016; Pos et al., 2019; Priebe et al., 2016; Velligan et al., 2015) out of the seven trials that were conducted after these novel tools were introduced reported them as secondary outcome measures. Future studies should continue this progress and use the up-to-date scales as primary outcome measures. This will also help to differentiate more clearly between treatment effects on motivational and expressive negative symptoms.

Moreover, the heterogeneity of the interventions tested across trials limits the generalizability of our findings. Other than for the two studies testing BPT based on the same treatment manual, the interventions for CBT and CR were diverse. Nevertheless, within each category (i.e., CBT, CR) the similarities certainly outweighed the differences, so that we are confident that our conclusions are valid for interventions that are CBT-based or CR-based, respectively.

An aspect that relates to this discussion of therapy contents is that only few programs to date specifically address a comprehensive set of mechanisms of negative symptoms (for a review of such mechanisms see Strauss and Cohen, 2017). Motivation and Enhancement Training (MOVE) is an integrative treatment approach in this regard, but has been tested in only one RCT so far that resulted in a moderate treatment effect (Velligan et al., 2015). Another integrative approach, the Positive Emotions Program for Schizophrenia (PEPS; Favrod et al., 2019; Nguyen et al., 2016) incorporates mindfulness-based techniques to supplement cognitive and behavioral group therapy contents.

Moreover, due to the small number of studies reporting comparable follow-up data, we were unable to include this aspect into our report. There is some evidence suggesting that treatment gains grow up to six months post treatments (Cella, Preti, Edwards, Dow, & Wykes, 2017; Favrod et al., 2019). However, one CBT-study in our sample showed that

treatment gains disappeared within six months (Pos et al., 2019). More studies are certainly needed that monitor the longevity of potential treatment gains.

Finally, some important limitations also arise from the central feature of our meta-analysis, namely that we only included studies that had offered treatment to patients with at least some minimum level of negative symptoms. As had to be expected, the number of studies fulfilling this criterion was low and so was the power of our analyses. This also prevented us from performing comprehensive moderator analyses. Others have pointed out the benefits of broader literature searches, which go beyond power issues and include the possibility of conducting network meta-analysis (Cella & Preti, 2017). Another viable approach to consider in future meta-analyses was employed by Turner and colleagues (2018) who, along a priori defined criteria, presented separate analyses progressing from a more to a less inclusive study selection.

It is also worth noting that even our strict inclusion criterion does not rule out the possibility that treatment effects are attributable to secondary sources of negative symptoms (cf. supplementary discussion in Fusar-Poli et al., 2015). Nevertheless, we consider our approach as an approximation to solving this “pseudo-specificity” issue because it assured that all included studies had at least focused on the treatment of relevant negative symptoms. We also want to emphasize that relatively simple adjustments to routine interview catalogues may help to exclude at least some sources of secondary negative symptoms, such as delusions. For instance, Pos and colleagues (2019) plainly asked their participants if their social withdrawal was due to delusions or hallucinations and did not count such behavior as negative symptoms.

Nevertheless, this meta-analysis was intended to help practitioners in selecting adequate psychological treatments for their patients with schizophrenia who also experience negative symptoms. Our approach reflects our assumption that practitioners will select a treatment that they believe has established efficacy within the patient group their patient belongs to (i.e. patients with schizophrenia who experience negative symptoms).

References

Bailer, J., Takats, I., & Schmitt, A. (2002). Individualisierte Kognitive Verhaltenstherapie bei schizophrenen Patienten mit Negativsymptomatik und sozialer Behinderung: II. Responderanalysen und Prädiktoren des Therapieerfolgs. *Verhaltenstherapie*, *12*(3), 192–203. https://doi.org/10.1159/000066570

Borenstein, M., Hedges, L. V., Higgins, J. P. T., & Rothstein, H. R. (2009). *Introduction to meta-analysis*. Chichester, U.K: John Wiley & Sons.

Buchanan, R. W. (2007). Persistent Negative Symptoms in Schizophrenia: An Overview. *Schizophrenia Bulletin*, *33*(4), 1013–1022. https://doi.org/10.1093/schbul/sbl057

Cella, M., & Preti, A. (2017). Psychosocial intervention for negative symptoms: a note on meta-analyses. *British Journal of Psychiatry*, *211*(02), 116. https://doi.org/10.1192/bjp.211.2.116

Cella, M., Preti, A., Edwards, C., Dow, T., & Wykes, T. (2017). Cognitive remediation for

negative symptoms of schizophrenia: A network meta-analysis. *Clinical Psychology Review*, *52*, 43–51. https://doi.org/10.1016/j.cpr.2016.11.009

Choi, K.-H., Jaekal, E., & Lee, G.-Y. (2016). Motivational and Behavioral Activation as an Adjunct to Psychiatric Rehabilitation for Mild to Moderate Negative Symptoms in Individuals with Schizophrenia: A Proof-of-Concept Pilot Study. *Frontiers in Psychology*, *7*, 1759. Retrieved from https://www.ncbi.nlm.nih.gov/pmc/articles/PMC5107574/pdf/fpsyg-07-01759.pdf

Favrod, J., Nguyen, A., Chaix, J., Pellet, J., Frobert, L., Fankhauser, C., … Bonsack, C. (2019). Improving Pleasure and Motivation in Schizophrenia: A Randomized Controlled Clinical Trial. *Psychotherapy and Psychosomatics*, 1–12. https://doi.org/10.1159/000496479

Fusar-Poli, P., Papanastasiou, E., Stahl, D., Rocchetti, M., Carpenter, W., Shergill, S., & McGuire, P. (2015). Treatments of Negative Symptoms in Schizophrenia: Meta-Analysis of 168 Randomized Placebo-Controlled Trials. *Schizophrenia Bulletin*, *41*(4), 892–899. https://doi.org/10.1093/schbul/sbu170

Horan, W. P., Kring, A. M., Gur, R. E., Reise, S. P., & Blanchard, J. J. (2011). Development and psychometric validation of the Clinical Assessment Interview for Negative Symptoms (CAINS). *Schizophrenia Research*, *132*(2–3), 140–145. https://doi.org/10.1016/j.schres.2011.06.030

Kirkpatrick, B., Fenton, W. S., Carpenter, W. T., & Marder, S. R. (2006). The NIMH-MATRICS Consensus Statement on Negative Symptoms. *Schizophrenia Bulletin*, *32*(2), 214–219. https://doi.org/10.1093/schbul/sbj053

Kirkpatrick, B., Strauss, G. P., Nguyen, L., Fischer, B. A., Daniel, D. G., Cienfuegos, A., & Marder, S. R. (2010). *The Brief Negative Symptom Scale : Psychometric Properties*. 4–9. https://doi.org/10.1093/schbul/sbq059

Klingberg, S., Wölwer, W., Engel, C., Wittorf, A., Herrlich, J., Meisner, C., … Wiedemann, G. (2011). Negative symptoms of schizophrenia as primary target of cognitive behavioral therapy: results of the randomized clinical TONES study. *Schizophrenia Bulletin*, *37 Suppl 2*(Suppl 2), S98-110. https://doi.org/10.1093/schbul/sbr073

Marder, S. R., & Galderisi, S. (2017). The current conceptualization of negative symptoms in schizophrenia. *World Psychiatry*, *16*(1), 14–24. https://doi.org/10.1002/wps.20385

Nguyen, A., Frobert, L., McCluskey, I., Golay, P., Bonsack, C., & Favrod, J. (2016). Development of the Positive Emotions Program for Schizophrenia: An Intervention to Improve Pleasure and Motivation in Schizophrenia. *Frontiers in Psychiatry*, *7*(13). https://doi.org/10.3389/fpsyt.2016.00013

Peralta, V., & Cuesta, M. J. (1995). Negative Symptoms in Schizophrenia: A Confirmatory Factor Analysis of Competing Models. In *Am J Psychiatry I* (Vol. 52). Retrieved from https://ajp.psychiatryonline.org/doi/pdf/10.1176/ajp.152.10.1450

Pos, K., Franke, N., Smit, F., Wijnen, B. F. M., Staring, A. B. P., Van Der Gaag, M., … Schirmbeck, F. (2019). Cognitive behavioral therapy for social activation in recent-onset psychosis: Randomized controlled trial. *Journal of Consulting and Clinical Psychology*, *87*(2), 151–160. https://doi.org/10.1037/ccp0000362

Priebe, S., Savill, M., Wykes, T., Bentall, R., Lauber, C., Reininghaus, U., … Röhricht, F. (2016).

Clinical effectiveness and cost-effectiveness of body psychotherapy in the treatment of negative symptoms of schizophrenia: a multicentre randomised controlled trial. *Health Technology Assessment*, *20*(11), 1–100. https://doi.org/10.3310/hta20110

Riehle, M., Jung, E., Wiesjahn, M., Mehl, S., Rief, W., & Lincoln, T. M. (2015). What's in an item? Predicting social outcomes in schizophrenia spectrum disorders from the PANSS item "Poor Rapport." *Schizophrenia Research*, *168*(1–2), 593–594. https://doi.org/10.1016/j.schres.2015.08.026

Savill, M., Banks, C., Khanom, H., & Priebe, S. (2015). Do negative symptoms of schizophrenia change over time? A meta-analysis of longitudinal data. *Psychological Medicine*, *45*(08), 1613–1627. https://doi.org/10.1017/S0033291714002712

Strauss, G. P., & Cohen, A. S. (2017). A Transdiagnostic Review of Negative Symptom Phenomenology and Etiology. *Schizophrenia Bulletin*, *43*(4), 712–729. https://doi.org/10.1093/schbul/sbx066

Strauss, G. P., Nuñez, A., Ahmed, A. O., Barchard, K. A., Granholm, E., Kirkpatrick, B., … Allen, D. N. (2018). The Latent Structure of Negative Symptoms in Schizophrenia. *JAMA Psychiatry*, *75*(12), 1303. https://doi.org/10.1001/jamapsychiatry.2018.2475

Turner, D. T., McGlanaghy, E., Cuijpers, P., van der Gaag, M., Karyotaki, E., & MacBeth, A. (2018). A Meta-Analysis of Social Skills Training and Related Interventions for Psychosis. *Schizophrenia Bulletin*, *44*(3), 475–491. https://doi.org/10.1093/schbul/sbx146

Velligan, D. I., Roberts, D., Mintz, J., Maples, N., Li, X., Medellin, E., & Brown, M. (2015). A randomized pilot study of MOtiVation and Enhancement (MOVE) Training for negative symptoms in schizophrenia. *Schizophrenia Research*, *165*(2), 175–180. https://doi.org/10.1016/j.schres.2015.04.008
